# The CO Tolerance of Pt/C and Pt-Ru/C Electrocatalysts in a High-Temperature Electrochemical Cell Used for Hydrogen Separation

**DOI:** 10.3390/membranes11090670

**Published:** 2021-08-31

**Authors:** Leandri Vermaak, Hein W. J. P. Neomagus, Dmitri G. Bessarabov

**Affiliations:** 1 HySA Infrastructure Centre of Competence, Faculty of Engineering, North-West University, Potchefstroom 2520, South Africa; 2 Centre of Excellence in Carbon Based Fuels, Faculty of Engineering, School of Chemical and Minerals Engineering, North-West University, Potchefstroom 2520, South Africa; Hein.Neomagus@nwu.ac.za

**Keywords:** electrochemical hydrogen separation, proton exchange membrane, polybenzimidazole membrane, cyclic voltammetry, electrochemical active surface area, CO poisoning, Pt-based catalysts

## Abstract

This paper describes an experimental evaluation and comparison of Pt/C and Pt-Ru/C electrocatalysts for high-temperature (100–160 °C) electrochemical hydrogen separators, for the purpose of mitigating CO poisoning. The performances of both Pt/C and Pt-Ru/C (Pt:Ru atomic ratio 1:1) were investigated and compared under pure hydrogen and a H_2_/CO gas mixture at various temperatures. The electrochemically active surface area (ECSA), determined from cyclic voltammetry, was used as the basis for a method to evaluate the performances of the two catalysts. Both CO stripping and the underpotential deposition of hydrogen were used to evaluate the electrochemical surface area. When the H_2_/CO gas mixture was used, there was a complex overlap of mechanisms, and therefore CO peak could not be used to evaluate the ECSA. Hence, the hydrogen peaks that resulted after the CO was removed from the Pt surface were used to evaluate the active surface area instead of the CO peaks. Results revealed that Pt-Ru/C was more tolerant to CO, since the overlapping reaction mechanism between H_2_ and CO was suppressed when Ru was introduced to the catalyst. SEM images of the catalysts before and after heat treatment indicated that particle agglomeration occurs upon exposure to high temperatures (>100 °C)

## 1. Introduction

Electrochemical hydrogen separators (EHSs) show great potential for future hydrogen-based energy scenarios. The main advantage that EHSs offer is the simultaneous compression and purification of hydrogen for fuel cell (FC) application and storage purposes [1,2,3]. Perfluorinated sulfonic acid (PFSA) membranes (mainly Nafion-based), typically operated at 60–80 °C, are generally used as proton exchange membranes (PEMs) due to their high proton conductivity, mechanical integrity, and chemical stability [4]. Pt-based materials are still the most favored electrocatalysts, although the focus of much current research is on alternative catalysts. Pt-based electrocatalysts offer effectiveness, high chemical stability, high current density, and good activities for hydrogen oxidation/evolution reactions in EHSs and oxygen reduction reactions in FCs [5,6]. However, Pt does have some disadvantages: resources are limited, it is costly, and Pt is prone to poisoning/deactivation [6,7,8]. Essentially, Pt is poisoned by low concentrations of carbon monoxide (>10 ppm) [4,7,9], and an explanation of this behavior is provided by the two competing reactions of H_2_ and CO adsorption [10]:

(1) $$2Pt+H_{2}\to 2Pt-H_{ads}$$

(2) $$Pt+CO\to Pt-CO_{ads}$$

Based on these equations, the performance deterioration of the cell is caused by CO adsorption onto the Pt surface, blocking many of the Pt sites used for the adsorption and oxidation of hydrogen [7]. However, the CO tolerance of Pt-based catalysts can be enhanced either by increasing the operating temperature or by employing bimetallic catalysts [9,11]. To this end, high-temperature (HT)-PEMs (operating temperatures 100–200 °C) with concentrated phosphoric acid (PA) as electrolyte have been developed to overcome the limitations associated with LT PFSA membranes [6,12,13], and platinum alloy catalysts such as Pt-Ru have been developed and implemented in low-temperature (LT) Nafion-based membrane EHSs and FCs [11,14]. Take note that for the purpose of this study in the field of electrochemical hydrogen separation, HT and LT refers to the temperature range 100–200 °C and <100 °C, respectively. Starting with the latter, Ru is known to weaken the binding of Pt–CO whilst strong binding of Ru–CO occurs, and Ru atoms adsorb oxygen-containing atoms, oxidizing CO to CO_2_, as is seen in FCs [15]. Nevertheless, when the anode is operated at a potential high enough to oxidize CO to CO_2_, it could lead to substantial losses in the cell efficiency [16].

In terms of HT-PEMs, polybenzimidazole (PBI) membranes are the most widely studied HT-PEMs to date; they show great proton conductivity due to their high PA doping capabilities [13]. PA-doped PBI membranes conduct protons well, even under anhydrous conditions, which significantly simplifies the electrochemical system because the humidification steps are eliminated, hence reducing water management issues [11]. Further advantages include fast electrode kinetics, enhanced mass transport, higher tolerance to impurities such as CO (up to 3 vol. %), and better sustainability [4,17,18,19]. Moreover, an increase is expected in the transport of gases through the flow fields and their diffusion in the gas diffusion layer due to the absence of liquid water. This, in turn, allows for simplified flow field designs and improved interaction between reactants and reaction sites [11]. However, the higher Pt loadings required, due to the free nature of the acid, undermine these advantages [11]. In efforts to reduce costs, whilst maintaining efficiency, noble metals are combined with carbon particles [8]. To further reduce the costs, highly porous nanostructured catalysts, known as Pt black, have been used in the electrodes. These catalysts are used to maximize the surface area and to attain ideal contact between the gas phase, electrode (electrically conductive), and the electrolyte (ion-conductive), for optimal performance [13,20]. 

Cyclic voltammetry (CV) is a very popular technique to determine the electrochemical active surface area (ECSA) of the electrode. The ECSA is a key performance parameter that relates to the catalytic activity of the electrode (e.g., the catalyst/electrode degradation) [13,21], especially when the electrode has a low Pt content [22,23]. In general, the ECSA is used as a measure for assessing electrocatalyst utilization in electrochemical cells, e.g., in FCs [24].

For carbon-supported Pt, two common methods exist to measure the ECSA: hydrogen underpotential deposition (H_upd_) and CO stripping (COS) [25]. The former is a very popular method and has been widely applied (from rotating disk electrode (RDE) systems to electrochemical cells, where the electrode is integrated with the membrane electrode assembly (MEA)) [22,23,26]. However, despite being widely implemented, several concerns related to the accuracy of these methods have been reported earlier [22,27]. These concerns include the H_2_ adsorption/desorption characteristics that may be masked by the carbon (e.g., the double-layer charging behavior of surface groups on carbon) [21] and severe corrosion of Pt nanoparticles at potentials >1.1 V due to oxidation of the carbon support [25]. When operating at >100 °C, additional concerns arise, such as (i) the strongly adsorbed PA anions from the dissociation of concentrated PA (mainly H_2_PO_4_^−^) that may inhibit the adsorption of hydrogen and (ii) the high Faradic currents from the hydrogen evolution reaction (HER) (the electrochemical reduction of H^+^ ions), which leads to the formation of H_2_ molecules that can superimpose the pseudocapacitive currents from the hydrogen underpotential deposition layer [28]. Improved methods to obtain baselines for the H_upd_ signal and the COS peak have been developed to allow reproducible and consistent values for Pt-based ECSA [29]. In the case of Pt alloys, Becknell et al. [30] suggest using both COS and H_upd_ alongside each other for ECSA evaluations to avoid the underestimation of the specific areas of the electrodes, associated with the overestimation of the surface area-normalized catalytic activity.

In open literature, in situ measurements (i.e., MEA) of the electro-oxidation kinetic parameters of CO adsorbed on Pt-based catalysts are rarely reported due to the complex overlapping of the different oxidation mechanisms. Ex situ measurements (e.g., RDE) are preferred and either measured from electrode in solution mechanisms [31] or deduced from fitting a model to the CO performance loss data [32]. It is common for catalytic properties measured from ex situ measurements (e.g., RDE) to disagree with those measured in MEAs [22]. Therefore, in order to develop and design improved electrocatalytic materials, particularly for FCs and related processes, the fundamental aspects of CO oxidation on real electrocatalysts need to be understood. Since ex situ and in situ results vary greatly, this can only be achieved when the in situ methods are applied. Therefore, the current investigation focuses solely on in situ measurements. 

As previously mentioned, the CO tolerance of an electrode can be increased by either Pt-Ru or high-temperature operation. To the knowledge of the authors, no studies have been conducted where CO poisoning mitigation has been studied by a combined approach of Pt-Ru and HT operation. Therefore, a combined approach that includes both HT-PEMs and Pt-Ru catalysts is now proposed. To effectively evaluate the practicality of the proposed method, the performance of a Pt-Ru/C was compared with that of a Pt/C. The same PA-doped PBI membranes were used for both catalysts, in the temperature range 80–160 °C. COS scans are typically performed with CO/N_2_ gas mixtures; however, since the focus of this study is on electrochemical hydrogen separation, a H_2_/CO gas mixture (2% CO, balance H_2_) was used. Three types of ECSA methods were considered and compared: H_upd_-based ECSA, COS-based ECSA, and H_upd_/COS-based ECSA [33]. The three ECSAs were evaluated from COS and H_upd_ stripping steps applied. The COS-based ECSA determination proved to be ineffective in the presence of H_2_ due to the complex overlapping of the oxidation mechanisms; therefore, the novel CO-H_upd_ peak was used to estimate the ECSA from the COS-CV data. For HT-PEM electrochemical cells, the distribution of the electrolyte in the MEA determines the availability of the triple phase boundary and, therefore, influences the ECSA. The mobility of the PA results in lower catalyst utilization and, ultimately, a lower ECSA [13]. This is expected to have an unavoidable effect on the experimental ECSA results. However, this effect was considered to be minimal.

## 2. Experimental

### 2.1. Experimental Rig

Three gas mixtures were used to conduct the experiments. Pure H_2_ was used to characterize the performance of both the Pt/C and Pt-Ru/C MEAs and to determine the H_upd_-based ECSA. A 2 vol. % CO (balance H_2_) gas mixture was used for the COS-based ECSA, for both the Pt/C and Pt-Ru/C MEAs. Dry N_2_ was used to flush the cathode compartment and to purge the system. All gases were stored in high-pressure gas cylinders, regulated by pressure regulators, and connected to the rig through small-diameter (1/4’’) plastic tubing. Mass flow controllers (Brooks Delta Smart II) were used to regulate the flow of the feed streams. The mass flow controllers were controlled making use of LabVIEW software. 

All experiments were carried out at atmospheric pressure. The cell operating temperature was varied between 80 and 160 °C and maintained by means of two Watlow FIREROD heating rods (one at the anode side and one at the cathode side). A K-type thermocouple (anode side) and PID controller (DCL-33A) were incorporated into the cell. Furthermore, the cell was enclosed in a heat insulation jacket to reduce heat losses. The temperature at the center of the anode back plate was taken as the cell temperature. 

All experiments were conducted in hysteretic order as per assessment of the performance deterioration during experimentation. The flow rates herein reported are all given in normal milliliters per minute (mL_n_/min), with 0 °C and 1 bar taken as reference. All potentials reported herein refer to the reversible hydrogen electrode (RHE) at the specified temperature. 

### 2.2. Electrochemical Cell

**Figure 1:**
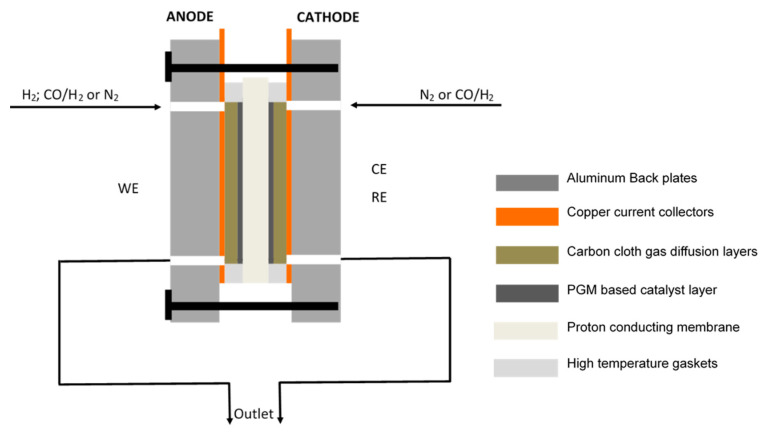
Schematic of the electrochemical cell.

A two-electrode system was used, with the anode acting as the working electrode (WE), whilst the cathode acts as both the counter electrode (CE) and reference electrode (RE), as illustrated in Figure 1. Here, the cathode also acts as an RHE (reference electrode). A clear description of the RHE is given by Jerkiewicz [34].

Experiments were carried out using a single, square-shaped, off-the-shelf electrochemical cell with an active area of 25 cm^2^ (Fuel Cell Technologies, Inc., Albuquerque, NM, USA) making use of a multipath serpentine flow field design for gas distribution. The cell comprised a proton-conducting membrane, placed between two pieces of carbon cloth (acting as gas diffusion layers); copper current collectors, sealed off with high-temperature PFA gaskets (used to compress the MEA); and aluminium back plates used for assembly.

The cell was compressed using a torque wrench to ensure a uniform pressure distribution over the compressed MEA and to minimize the electrochemical and thermal resistance of the gas diffusion layers. The assembly torque for the eight bolts was 6 Nm. Identical PBI membranes were tested, with different anode catalysts (one Pt/C and the other Pt-Ru/C). The cathode catalyst for the two membranes was exactly the same, Pt/C. The total metal loadings for both Pt and Pt-Ru were 2 mg cm^−2^.

### 2.3. Performance Evaluation: Pure Hydrogen

Experiments were first conducted using pure hydrogen. The effect of temperature on the performance of the cell was investigated by varying the temperature between 100 and 160 °C, in 20 °C steps, for hydrogen flow rates of 50–400 mL_n_/min, in 50 mL_n_/min intervals. Polarization curves were drawn for each temperature and flow rate, commencing at the lowest temperature and flow rate. The flow rate was then raised to the second lowest flow rate, and the process was repeated until the highest flow rate was reached, after which the temperature was again raised by 20 °C and the process repeated once again. This was done until a limiting current was reached or until the voltage limit (1.1 V) was reached, after which the run was terminated. The volumetric flow rate of the permeate was recorded for each set current density at the respective temperatures and flow rates, making use of soap flow meters. 

To determine the resistance of the MEA in situ, electrochemical impedance spectroscopy (EIS) was performed in constant current/galvanostatic mode (1 A), frequency range 100 kHz to 0.1 Hz, at 100–160 °C, in hydrogen pumping mode. For feed streams containing impurities, the hydrogen flow rate was held constant for all EIS experiments at 100 mL_n_/min H_2_. The AC current amplitudes were a standard 10% of the applied direct current [9]. A Nyquist plot of the impedance spectrum was generated.

### 2.4. ECSA Evaluation Methods

#### 2.4.1. H_upd_: ECSA Evaluation

Voltammetric measurements were conducted using dry H_2_ at the anode (the WE). The cathode was flushed with dry N_2_. The third cycle comprised a total of three CV scans, which were used to determine the H_upd_-based ECSA. These cyclic voltammograms (also abbreviated CVs for simplicity of discussion) were recorded with a scan rate of 50 mV s^−1^, and the upper and lower potential was chosen as 1.0 and –0.025 V vs. the RHE. 

#### 2.4.2. CO stripping: ECSA Evaluation

The CO experiments were conducted based on the following procedure: (i) The anode was purged with 2% CO in H_2_ (overall flow rate 100 mL_n_/min) for 10 min, with a constant electrode potential of 0.1 V vs. RHE. Successively, (ii) the monolayer of adsorbed CO was stripped off and a CV scan was conducted at 20 mV s^−1^, from 0.1 V vs. RHE to 1.0 V vs. RHE. Three cycles were performed. Then, (iii) the CO reference baseline experiments were executed according to the same procedure, the only difference being that the tests were performed in N_2_-saturated electrolyte (CO was not present in this experiment). Here, the potential was initially held at 0.1 V for 30 min, followed by a CV scan at 20 mV s^−1^.

#### 2.4.3. Method Development

For the application of all CV methods, it is important to define a baseline for the determination of the H_upd_ and CO oxidation charge. This is essential for reproducibility and achieving diagnostically conclusive results. Consequently, two additional parameters should be defined: the upper and lower potential limits. The methods considered to define the baselines are now described.

*H_upd_—ECSA _(Capacitive charge baseline)_*: In the capacitive charge analysis, the double layer region is used as the subtraction baseline for the hydrogen adsorption/desorption peaks [25]. The potential at the current density minimum before the hydrogen evolution peak is taken as the lower potential limit for the integration of the charges. The upper limit is the potential after the hydrogen adsorption/desorption peak. This baseline method is referred to as the ‘standard baseline’.

For improving the H_upd_ analysis, Mayrhofer et al. [29] have suggested that the CV currents obtained from the CO-saturated baseline be subtracted from the H_upd_ signal instead of the traditional standard baseline currents (N_2_-saturated baseline). The same method was also used by Benninger et al. [25]. However, both these references considered only ex situ application; therefore, the reliability of in situ measurements remains as yet unknown, and clarity is still required. The method used by both Mayrhofer et al. [29] and Benninger et al. [25] was nonetheless considered, but preliminary test revealed that it was not reliable, and it was therefore disregarded.

*COS—ECSA _(Ref. CV baseline)_*: The reference CV is used to obtain a correct integration baseline without the adsorption of CO. This is crucial, since contributions from sources other than CO oxidation are possible, and hence correction is required. Therefore, the reference CV peak is used as a baseline, where the integration boundaries can be defined as the potentials where the reference CV peak and the COS CV peak (cycle 1) intersect.

### 2.5. Experimental Procedure

During the electrochemical measurements, the cell temperature was controlled in the range 80–160 °C. As a basic characterization step, pure hydrogen was used. The operational sensitivities and performances were investigated for both Pt/C and Pt-Ru/C electrocatalysts. This was followed by the ECSA determination tests, where the experiments on the electrodes were organized as follows: First, the cleaning step was performed. The anode and cathode were completely purged with N_2_, after which the CE was purged with H_2_. To ensure that all contaminants on the WE were removed, potential cycles between –0.025 and 1 V vs. RHE were conducted for 30 cycles, at a scan rate of 50 mV s^−1^. In the case of Pt-Ru, preconditioning was completed within narrower potential limits, with the upper potential limit set to 0.8 V. This was done to avoid irreversible oxidation or possible Ru dissolution [35]. In the second step, referred to as the reference CV scan, N_2_ was fed to the cell, and the baseline for the CO-ECSA _Ref_. _CV baseline_ was obtained at the different temperatures. Following this, the H_upd_ CV scan was performed at 50 mVs^−1^. The final step was the actual COS measurement. The reason for this organization is that once CO is introduced into the system, the entire removal thereof is challenging, and then interference from the oxidation of residual CO is possible. Hydrogen separation of the gas mixtures was also carried out to determine, and compare, the separation efficiency of both Pt/C and Pt-Ru/C.

#### ECSA Calculation

Evaluation of the ECSAs of Pt- and Pt-based electrocatalysts firstly involves the measurement of the charge of the adsorbed species ($Q_{H}$ or $Q_{CO}$), determined from the CV scans:

(3) $$I=\frac{dQ}{dt}$$

(4) $$Q_{i}=\int_{t_{i}}^{t_{f}}Idt=\frac{1}{v}\int_{E_{i}}^{E_{f}}IdE$$

where $t_{i\;}$ is the time at which the hydrogen adsorption commences and $t_{f}$ is the moment at which the monolayer is completed; E and I depict the potential (*V*) and current (A), respectively; and ν is the applied scan rate (mV s^−1^). Gamry Echem Analyst software was used to determine the charge area for all the respective CV peaks. Accordingly, the charge was normalized using a surface area-specific charge of an ideal one-electron transfer 210 μC cm^−2^ (H_upd_) or two-electron transfer 420 μC cm^−2^ (CO). Using the surface area-specific charge, the H_upd_-based and COS-based ECSAs of the anode were calculated using the following equations [27,28,36,37]:

(5) $$ECSA_{\left(H\right)}\left(\frac{cm^{2}Pt}{mgPt}\right)=\frac{Q_{H}(\mu C)}{210\left(\frac{\mu C}{cm^{2}Pt}\right)\times L\left(\frac{mgPt}{cm^{2}}\right)\times 25\;cm^{2}}$$

(6) $$ECSA_{\left(CO\right)}\left(\frac{cm^{2}Pt}{mgPt}\right)=\frac{Q_{H}(\mu C)}{420\left(\frac{\mu C}{cm^{2}Pt}\right)\times L\left(\frac{mgPt}{cm^{2}}\right)\times 25\;cm^{2}}$$

where L refers to the catalyst load. 

## 3. Results and Discussion

### 3.1. Pure Hydrogen Experiments: Membrane Characterization

**Figure 2:**
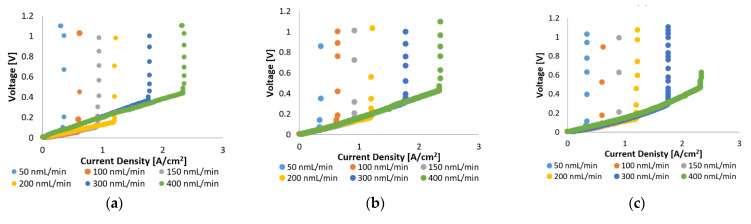

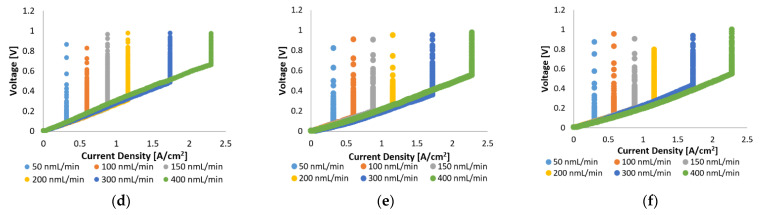
Top: Polarization curves for Pt/C at (**a**) 120 °C, (**b**) 140 °C, and (**c**) 160 °C. Bottom: Polarization curves for Pt-Ru/C at (**d**) 120 °C, (**e**) 140 °C, and (**f**) 160 °C.

In Figure 2, the voltage–current characteristics of the MEA with Pt/C and MEA with Pt-Ru/C are compared for pure hydrogen at various flow rates and cell operating temperatures (120–160 °C). The linear nature of the initial and intermediate sections of the plots indicate that the ohmic losses dominate the cell overpotential in this region, whilst the overall cell reaction rate dominates in the high current density region (horizontal lines). The latter is due to the depletion of reactants. The vertical lines in Figure 2 indicate the limiting current density, where most of the supplied hydrogen has been consumed; the limiting current density is dependent on the hydrogen supplied to the cell. Higher limiting current densities were reached when higher feed flow rates were used. This is explained by Faraday’s law, which states that the flow of hydrogen is directly proportional to the current.

In general, similar limiting current densities were reached for the two MEAs, although Pt/C exhibits better overall efficiency, indicated by the lower voltages that were achieved with Pt/C, compared to Pt-Ru/C, at the same current densities. Since the ohmic losses (defined as the sum of the contact resistance, electrical resistance, and proton conducting resistance) dominate the largest part of the polarization curves, the ohmic resistance is expected to be higher for the Pt-Ru/C since higher voltages are observed with this MEA. This was verified by the EIS experiments. 

**Figure 3:**
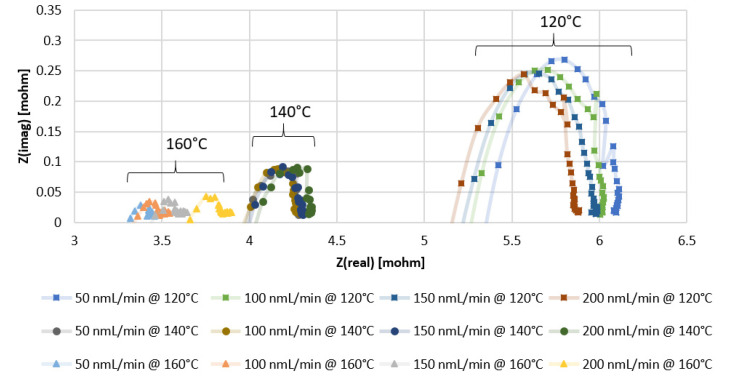
EIS results for PBI membrane (Pt/C catalyst) with pure hydrogen at different temperatures and flow rates.

**Figure 4:**
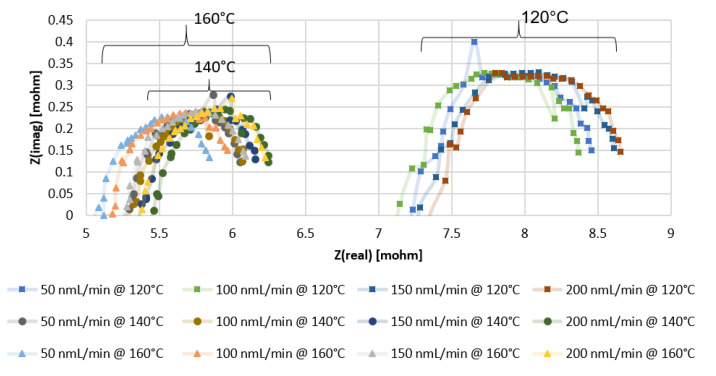
EIS results for PBI membrane (Pt-Ru/C catalyst) with pure hydrogen at different temperatures and flow rates.

From the Nyquist plots of the impedance data presented in Figure 3 and Figure 4, with the x-intercept resembling the ohmic resistance of the membrane, it can be observed that higher values are obtained with the MEA containing Pt-Ru/C. In Figure 3, the x-intercept values for the Pt/C MEA range between 5 and 5.5 mΩ at 120 °C, 3.9 and 4.1 mΩ at 140 °C, and 3.3 and 3.7 mΩ at 160 °C, whereas the ohmic resistance range of the Pt-Ru/C MEAs varies between 7 and 7.5 mΩ at 120 °C, 5.2 and 5.5 mΩ at 140 °C, and 5.1 and 5.4 mΩ at 160 °C (Figure 4). Further investigation of the EIS data indicates that the mass and charge transfer resistances (diameter of the impedance arc) are higher for the Pt-Ru/C MEA.

The higher ohmic resistance and the higher mass and transfer resistances seen for the Pt-Ru/C MEA may be explained by the distribution of the PA in the membrane, since the ohmic resistance is mainly related to the resistance of the electrolyte [38] and the ECSA should be directly related to the amount of the electrolyte contacting the catalyst [13]. The mobile nature of the PA results in lower catalyst utilization and ultimately lower ECSA, which may be the case with the Pt-Ru/C [13]. However, it is uncertain whether the higher resistances are due to the poor distribution of the acid inside the membrane, or whether it can be explained by the bifunctional mechanism involving Pt and Ru as active sites or an electronic effect of Ru on Pt. The opposite is seen in an alkaline environment: the oxidation of hydrogen on Pt is slow, and Pt-Ru alloys show exceptionally higher hydrogen oxidation reaction (HOR) activity [39]. No reports were found on the comparison of the HER and the HOR on Pt and Pt-Ru at high temperatures in an acidic environment. It is known, however, that hydrogen oxidation and evolution on Pt in acid are facile processes [40].

Furthermore, general observations can be made from the EIS data presented for both the Pt/C- and Pt-Ru/C-containing MEAs. Temperature is seen to enhance ohmic, mass, and charge transfer resistances. The reduction in the arc diameter at higher temperatures indicates that there is a slight improvement in mass transport and high-frequency charge transfer, as confirmed in literature [41]. Su et al. [42] reported that an increase in temperature enhances the cell performance due to an increase in the reaction and the mass transfer rate and a lower cell resistance, which is confirmed in this work. 

Another observation made is the influence of the hydrogen feed flow rate on the ohmic resistance and electrode kinetics. The ohmic resistance of both the Pt/C and Pt-Ru/C MEAs increases with the applied flow rate at temperatures ≥140 °C, whilst the electrode kinetics seems to be independent of the applied flow rate. At 120 °C, the resistance increase in terms of hydrogen feed flow rate (mL_n_/min) for Pt/C and Pt-Ru/C is 200 < 150 < 100 < 50 and 200 < 150 < 50 < 100, respectively. Similarly, tests performed at 100 °C (not indicated in the graphs) reveal the resistance dependence on the feed flow rate (in mL_n_/min) for both MEAs to be 200 < 150 < 100 < 50. According to the literature, excess fuel during EIS should minimize the mass transport resistance [18]. This was partly confirmed in this work—this was only observed at lower temperatures, where the resistance was seen to decrease with an increase in the fuel supply.

**Figure 5:**
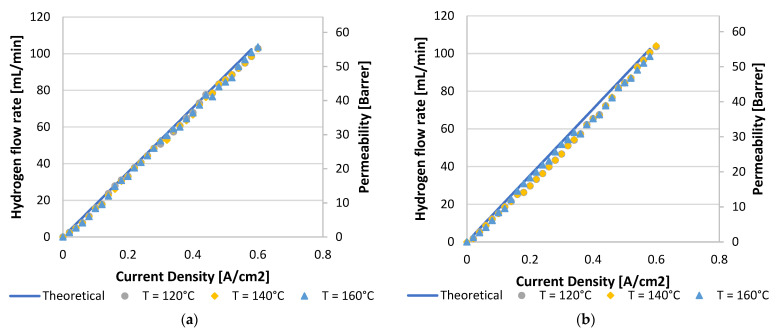
Hydrogen flux and permeation rate for a hydrogen feed flow rate of 100 mL_n_/min for (**a**) Pt/C MEA and (**b**) Pt-Ru/C MEA.

To conclude this section, the hydrogen flux measured with a bubble flow meter indicated that nearly Faradic flow rates were achieved throughout, indicating that no major system or cell leakages were present. Curves for a hydrogen feed flow rate of 100 mL_n_/min are demonstrated in Figure 5. Here, the theoretical line illustrates the law of Faraday. 

#### 3.1.1. Hupd-Based ECSAs

**Figure 6:**
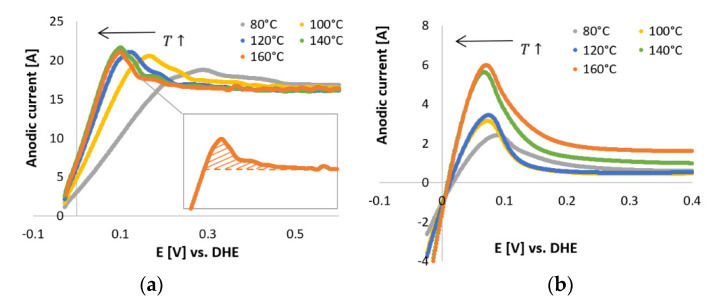
In situ CVs performed in N_2_-saturated electrolyte (PA) with a scan rate of 50 mV s^−1^. (**a**) Pt/C. (Inset) The filled area represents the charge related to the H_upd_ peak after applying the standard baseline correction at 160 °C. (**b**) Pt-Ru/C.

The CV scans obtained for the N_2_-saturated electrolyte for Pt/C and Pt-Ru/C are plotted in Figure 6. As the temperature increased, the H_upd_ peak shifted to the left (lower potential region), for both Pt/C and Pt-Ru/C. The shift was more significant in the case of Pt/C. This is indicative of the temperature dependency of the H_upd_ peak, which also influences the size and shape of the peak. For both Pt/C and Pt-Ru/C, the peak height increased with temperature, whilst the peak width appeared to become narrower. Broader smaller peaks are observed at 80 °C, whilst sharper narrower peaks are evident at 160 °C. 

**Figure 7:**
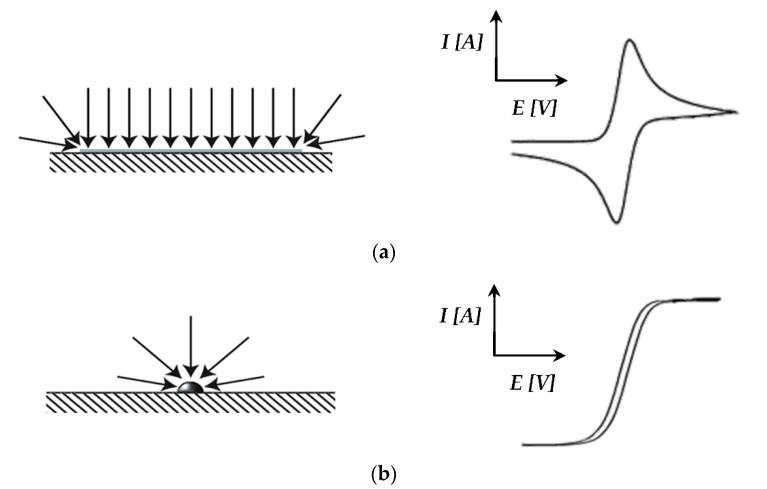
(**a**) Macroelectrode: planar diffusion with the resulting CV curve shape; (**b**) Micro/nanoelectrode: convergent/circular diffusion with the resulting CV curve shape (based on Banks et al. [43]).

The capacitive currents for the Pt/C catalyst (Figure 6a) were far above the zero current region, and higher currents were observed compared to the Pt-Ru/C catalyst. This can possibly be explained by the diffusion characteristics of the nanoparticles, where circular/convergent diffusion is present [43] (see Figure 7). The effect of convergent/circular diffusion poses the benefit of improvements in mass transport to an extent where the current density is greater than that of a macroelectrode under planar diffusion [43]. This was evident for both Pt/C and Pt-Ru/C, since the anodic current peaks are in the ranges of approximately 16–22 A and 2–6 A, respectively, whereas bulk current values are commonly expressed on a μA or mA scale [23,25,28,29]. Another point to consider is the fact that the system that was used is a two-electrode in situ MEA and not a typical three-electrode ex situ set-up. As mentioned earlier, MEA measurements are rarely performed, whereas RDE studies are abundant. The values obtained from MEAs vary greatly from those obtained by RDE systems. According to Garrick et al. [22], this is due to the lack of stability of highly active catalysts [44]. As the catalyst ages in an MEA, changes in the size, composition, and surface structure of the catalyst occur [22,45]. Other variables, such as the gas distribution, contact resistance between the cell components, catalyst support corrosion, PA concentration, and acid leaching/acid distribution should not be disregarded and could potentially also influence the CV scans. 

Results for the ECSA estimation by means of the standard baseline method for Pt/C and Pt-Ru/C are tabulated in Table 1 and Table 2, respectively (ECSA data column highlighted in grey). The tables show that the estimated ECSA values, in cm^2^ (mg catalyst)^−1^, for Pt/C and Pt-Ru/C are similar, with Pt/C exhibiting slightly higher ECSA values than Pt-Ru/C. 

**Figure 8:**
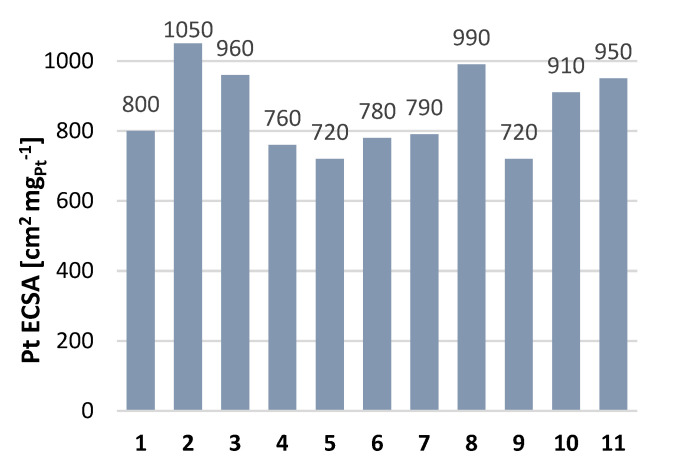
Pt ECSA values for a ~50 wt. % Pt/C standard (TKK-TEC-10E50E). Nafion indicates Nafion ionomer 1: Water/IPA/Nafion [47], 2: water/IPA/Nafion (optimized) [48], 3: water/Nafion film [49], 4: water [50], 5: water/Nafion film [51], 6: water/Nafion film [52], 7: water/ethanol/Nafion [53], 8: water/IPA/Nafion [54], 9: IPA/Nafion [55], 10: water/IPA/Nafion [56], 11: water/IPA/Nafion [57]. (reproduced from Garsany et al. [58]).

The ECSA values obtained are comparable to the ECSA value obtained for LT Nafion membranes, for 50 wt. % Pt/C (see Figure 8). For both Pt/C and Pt-Ru/C, the apparent ECSA increased with temperature. This may be explained by enhanced electrode kinetics [4,17,19,46].

#### 3.1.2. CO-Based ECSAs

**Figure 9:**
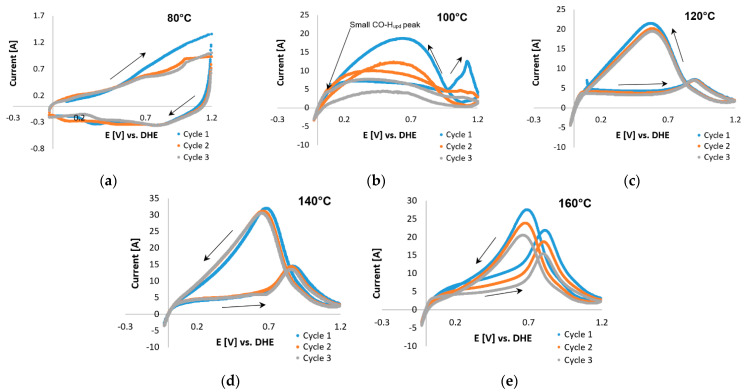
COS CVs of the Pt/C catalyst, 2% CO (balance hydrogen), performed with a scan rate of 20 mV s^−1^ with potential limits −0.025 and 1.2 V vs. RHE at (**a**) 80 °C, (**b**) 100 °C, (**c**) 120 °C, (**d**) 140 °C, and (**e**) 160 °C.

**Figure 10:**
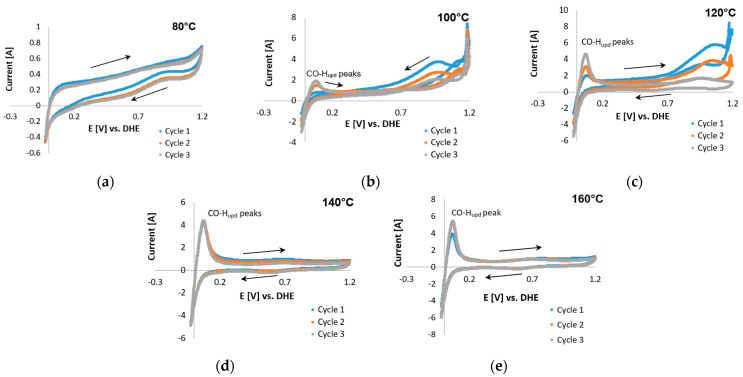
COS CVs of the Pt-Ru/C catalyst, 2% CO (balance hydrogen), performed with a scan rate of 20 mV s^−1^ potential limits −0.025 and 1.2 V vs. RHE at (**a**) 80 °C, (**b**) 100 °C, (**c**) 120 °C, (**d**) 140 °C, and (**e**) 160 °C.

The CO-stripping CV curves for Pt/C and Pt-Ru/C in the presence of H_2_ (98 vol. %) are presented in Figure 9 and Figure 10, respectively. The absence of peaks at 80 °C (Figure 9a) indicates that the Pt catalytic sites were deactivated by CO. This was also seen in the case of Pt-Ru/C at 80 °C (Figure 10a), which means that a large amount of the catalytic surface is blocked by CO. This is in agreement with literature; the CO tolerance for LTs (70–80 °C) is 50 ppm maximum [6]; since 2 vol. % CO is used to test the CO-based ECSAs, the catalytic sites become deactivated. Peak formation was observed at 100 °C (Figure 9b), with one large peak seen in the first potential cycle (forward direction) at ~1.1 V. In the succeeding cycles, this peak disappeared, which results from the removal of the monolayer of adsorbed CO. Although the entire peak had disappeared by cycle 3, only a small CO-H_upd_ peak was formed, which indicated that a large number of the catalytic sites may still be deactivated by CO. Typical CO oxidation potentials on Pt were reported to be around 0.5–0.8 V vs. RHE [25,28], which is in disagreement with the peak potential observed here in cycle 1. 

**Figure 11:**
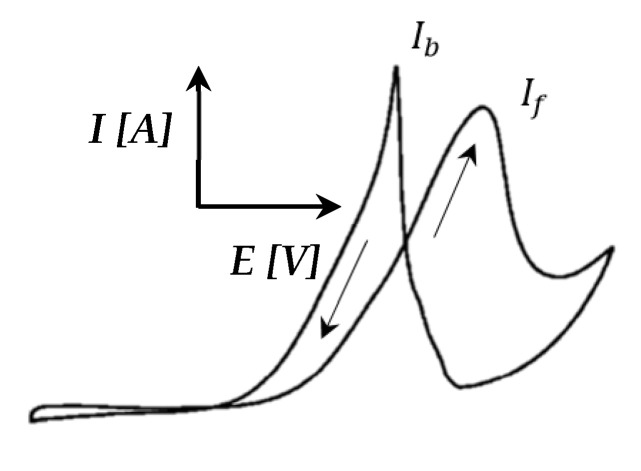
CV illustrating the reverse (*I_b_*) and forward (*I_f_*) current peaks (adapted from Yin et al. [59]).

Furthermore, a peak was seen in the reverse direction. This was repeated at higher temperatures. This peak became sharper and seemed to shift to lower potentials as the temperature was increased beyond ≤120 °C. The same peak was also visible in the CVs of Pt-Ru/C (Figure 10) at 100–120 °C, but it decreased as the temperature increased. Only CO-H_upd_ peaks were visible at 140–160 °C. Similar behavior has been reported for the methanol electro-oxidation reaction [52,59], where the CO tolerance of Pt-based electrocatalysts was compared by means of the peak current ratio in the reverse (*I_b_*) and forward (*I_f_*) directions, as illustrated in Figure 11. 

Both Sheng et al. [52] and Yin et al. [59] have studied the frequently invoked method to mitigate CO poisoning by introducing a foreign metal (X) to Pt, forming a Pt alloy, to facilitate the removal of adsorbed CO on the Pt surface, either by a bifunctional mechanism or by an electronic effect. Sheng et al. [52] studied Pt-Ru/C, whilst Yin et al. [59] studied Pt-Au. The current ratio criterion implemented in both these papers stated that the smaller the $I_{b}/I_{f}$ ratio of an electrocatalyst is, the greater the improvement in its CO tolerance and catalytic activity is [52].

Manoharan and Goodenough [60] speculated that the $I_{b}$ peak is associated with residual carbon species, rather than originating from the newly oxidized chemisorbed species. This means that $I_{b}$ presumably does not have the same chemical origin as $\;I_{f}$. However, the accuracy of this method was considered ambiguous for the curves considered, since the forward and reverse peaks seemed to be related in some manner. This conclusion was drawn from the Pt/C data, which indicated that a decrease in one peak (forward/reverse) is accompanied by a decrease in the other peak (reverse/forward).

Although the origin of the reverse peak is vague, the peaks found in the potential region 0.7–1.2 V for Pt-Ru/C may possibly be related to CO adsorption on the catalyst, since a decrease in these peaks seems to be directly related to an increase in the CO-H_upd_ peaks. This increase indicates that as the CO-related peaks decrease, the Pt sites become reactivated for hydrogen adsorption to take place, and hence a hydrogen peak emerges. Furthermore, since the forward current peak does not decrease with temperature, and it seemingly increases as the temperature is increased, the assumption was made that this peak originated from side reactions (as reported by Engl et al. [28]). Two possible side reactions were identified: one being the complex overlap of mechanisms and the other presumably originating from the oxidation of the carbon support [25]. Both these reactions were considered and investigated. 

**Figure 12:**
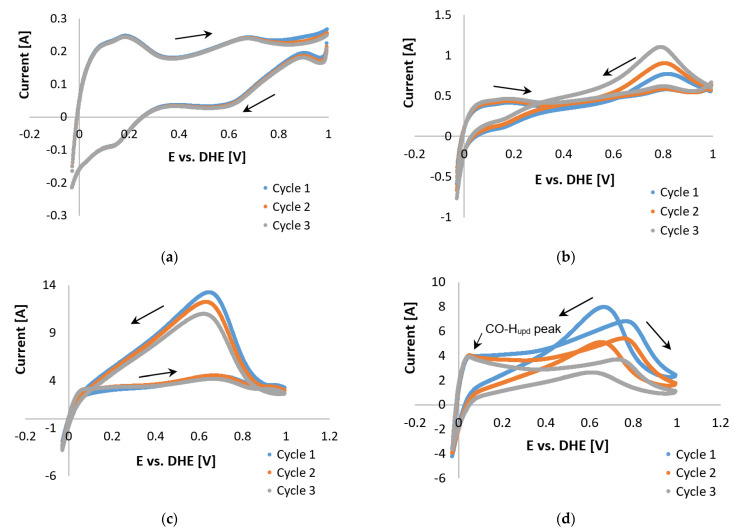
COS CVs of the Pt/C catalyst, 2% CO (balance hydrogen), performed with a scan rate of 20 mV s^−1^ with potential limits −0.025 and 1 V vs. RHE at (**a**) 100 °C, (**b**) 120 °C, (**c**) 140 °C, and (**d**) 160 °C.

Commencing with the latter, the Pt nanoparticle catalyst supported on high-surface-area carbon suffers from severe corrosion at potentials >1.1 V. It is for this reason that Zhang et al. [21] suggested that the anodic limit be set to <1.0 V vs. RHE. Following this suggestion, Pt/C COS scans in the presence of H_2_ and small amounts of H_2_O (from dilute PA) were repeated with an upper potential limit of 1 V vs. RHE. The curves at 100 °C were similar in shape to those reported by Schneider et al. [24] for pure hydrogen in a N_2_-saturated electrolyte, suggesting that the CO peak is not present. The forward peak disappeared almost completely at 120 and 140 °C and was once again visible at 160 °C (Figure 12). At 160 °C, the forward and reverse peaks decreased with each subsequent cycle, along with the CO-H_upd_ peak formation observed in cycle 3. The H_upd_ peaks were considered insignificant in terms of size. The reverse peak disappeared almost completely, which is to be expected because CO is no longer adsorbed onto Pt at 140–160 °C [6]. However, according to Garrick et al. [22], the HT suppresses the COS-based ECSA more than the H_upd_-based ECSA, due to the shift in the equilibrium of CO adsorption/desorption on the Pt surface. 

Upon comparing the results in Figure 9e and Figure 12d, the possible oxidation of the carbon support was observed when the potential limit was set to >1 V. The peaks in Figure 9d are much larger, yet both CVs do show improvement (decrease in peak size) with each cycle. Results observed in Figure 12 led to the conclusion that oxidation of the carbon support, although not completely disregarded, was not the origin of the reverse peaks. Therefore, the theory of overlapping mechanisms was investigated. A variety of reaction possibilities was considered, which basically include the assumption that *CO* is either reduced [61] or oxidized [62].

First, the latter is discussed. From a mechanistic point of view, the adsorbed *CO* is believed to be electrochemically oxidized by an oxygen-containing species (generally *OH*) [27,63] as follows:

(7) $$H_{2}O+Pt↔Pt-OH_{ads}+H^{+}+e^{-}$$

(8) $$Pt-CO_{ads}+Pt-OH_{ads}\to CO_{2}+H^{+}+e^{-}+2Pt$$

In the case of bimetallic catalysts (e.g., Pt-Ru) the reaction is suggested to change in the following manner:

(9) $$X+H_{2}O\to X-OH_{ads}+H^{+}+e^{-}$$

(10) $$Pt-CO_{ads}+X-OH_{ads}\to Pt+X+CO_{2}+H^{+}+e^{-}$$

where *X* denotes the foreign metal added to *Pt*. 

In the case of PEM-FCs, water is the only possible oxygen source, due to the low partial pressure of oxygen. Here, the activation of the water molecule is considered imperative for the oxidation of adsorbed CO on Pt-based electrocatalysts. However, although water is considered an important factor for CO electro-oxidation, most studies on the oxidation of CO_ads_ have been conducted in aqueous electrolyte (e.g., dilute H_2_SO_4_ or HClO_4_ solution). Since the electrolyte used consists of 85 wt. % PA, diluted in water, the water present was considered to be sufficient for the oxidation of CO.

**Figure 13:**
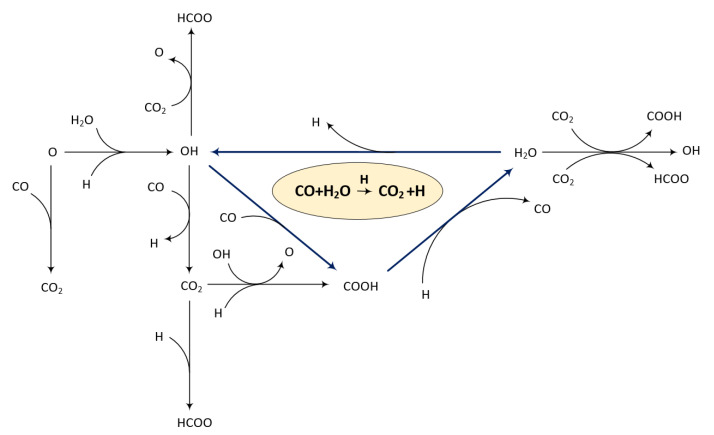
The reaction mechanisms of CO oxidation on Pt in the presence of H_2_ and water.

In other studies, the presence of H_2_ had promotional effects on CO oxidation similar to H_2_O; H_2_ can be oxidized to H_2_O or hydroxyl (OH) groups [64,65]. Since the anode feed gas consists of a mixture of hydrogen and CO, hydrogen is expected to have a promotional effect on the CO oxidation. Furthermore, coupling chemistries arise from the use of gas mixtures containing H_2_ and CO or CO, O, and H_2_O [66]. An example of such coupling is the production of oxygenates (e.g., COOH*) through a recombination reaction, as proposed by [66]:

(11) $$CO^{*}+OH^{*}\to COOH^{*}+*$$

with its subsequent decomposition

(12) $$COOH^{*}+OH^{*}\to CO_{2}^{*}+H_{2}O^{*}$$

or

(13) $$COOH^{*}+*\to CO_{2}^{*}+H^{*}$$

where the asterisk (*) resembles either an empty site or an adsorbed species. HCOO** also undergoes similar reactions. These coupling chemistries are important when considering surface reactions on Pt-based catalysts and need to be accounted for. Therefore, a surface reaction mechanism comprising three sub-mechanisms (CO oxidation, H_2_ oxidation, and CO-H_2_ coupling steps) was developed, as illustrated in Figure 13. The surface reaction mechanism was constructed based on the assumption that the PA used is stable and does not partake in any of the reactions. Moreover, the reactions were based on the surface reactions presented by [66]. 

The reaction mechanism presented in Figure 13 indicates that the surface reaction is more complex than previously thought. From this, the possibility of subsequent oxidation or the formation of intermediate carboxyl species (COOH) was identified, which may explain the reverse peak previously mentioned. The facile formation of intermediate carboxyl species has been reported previously, and subsequently confirmed, by experiments performed on Au surfaces [65]. 

Other reactions that should not be disregarded in the overall reaction mechanism include the electrochemical Fischer–Tropsch process [67] and electrochemical methanation. Both these reactions are based on the electrochemical reduction of CO in the presence of H_2_. The former involves the formation of valuable hydrocarbons, such as CH_4_ (methane), C_2_H_4_ (ethylene), and C_2_H_6_ (ethane) [67], whereas the latter involves the electrochemical conversion of CO to CH_4_. The electrocatalytic reduction of CO_2_ to CO and CH_4_ has been reported with the same device [61,68], showing the plausibility of electrochemical reduction with electrocatalytic cells. 

**Figure 14:**
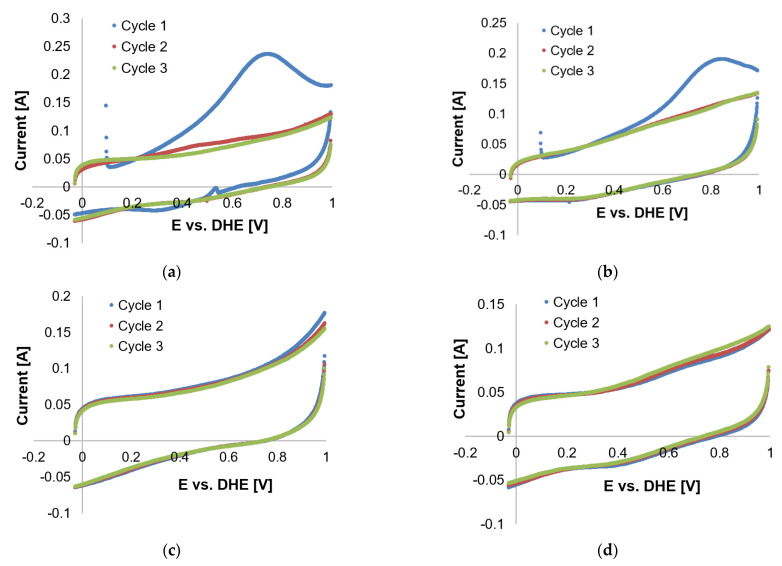
Cyclic voltammetry scans of 1% CO in N_2_ at (**a**) 100 °C, (**b**) 120 °C, (**c**) 140 °C, and (**d**) 160 °C.

Results of tests performed with 1 vol. % CO (balance N_2_, no H_2_ present) (see Figure 14) also serve as confirmation (for the coupling mechanisms in the presence of H_2_); no reverse peak is present and only one CO peak is seen in the first potential cycle. Therefore, no side reactions were evident in the absence of H_2_. No further attention was given to the identification of the side reactions because this aspect did not form part of the initial project scope.

Since the ECSA could not be evaluated from the CO peaks, the decision was made to evaluate the ECSA from the CO-H_upd_ peaks instead. The latter were considered to resemble the reactivated Pt sites. Since no CO-H_upd_ peaks were present in the CV scans performed on Pt/C, the ECSA values for only Pt-Ru/C could be analyzed (see Table 3). The values presented are not considered the real ECSA values, but are rather to be seen as an estimation. 

Table 3 clearly shows that, at the temperatures that were investigated in this study, the CO/H_upd_-based ECSA values increase with each consecutive cycle. Moreover, the ECSA values also increase as the temperature is increased (also seen previously for H_upd_-based ECSA values). However, the values obtained were lower than those depicted in Table 2. This could be explained by the change in acid distribution or the deactivation of Pt sites upon CO exposure. At temperatures <140 °C, other peaks originating from CO as a source were visible. Therefore, the lower ECSA values at these lower temperatures might be considered to be caused by CO poisoning. However, at temperatures ≥140 °C, no peaks other than H_upd_/CO are visible, and therefore CO poisoning was not considered to be the cause behind the lower ECSA values at temperatures >140 °C.

**Figure 15:**
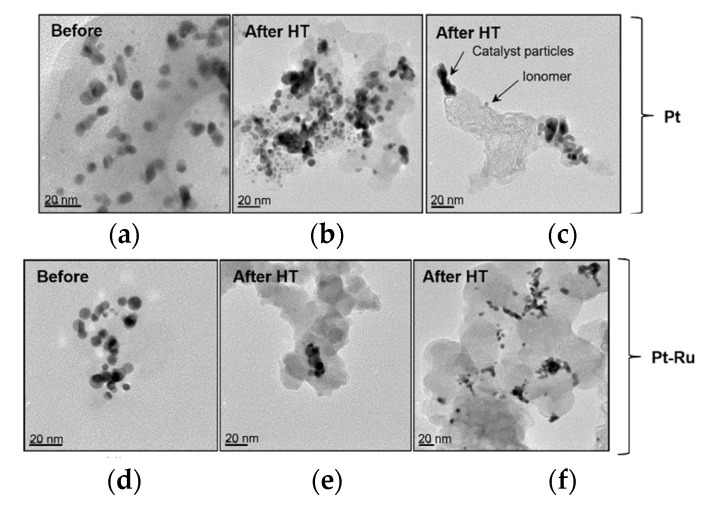
TEM images of Pt/C and Pt-Ru/C catalysts before and after CO and high-temperature exposure (HT): (**a**) Pt; (**b**) Pt-HT, (**c**) Pt-HT, (**d**) Pt-Ru, (**e**) Pt-Ru-HT, and (**f**) Pt-Ru-HT.

**Figure 16:**
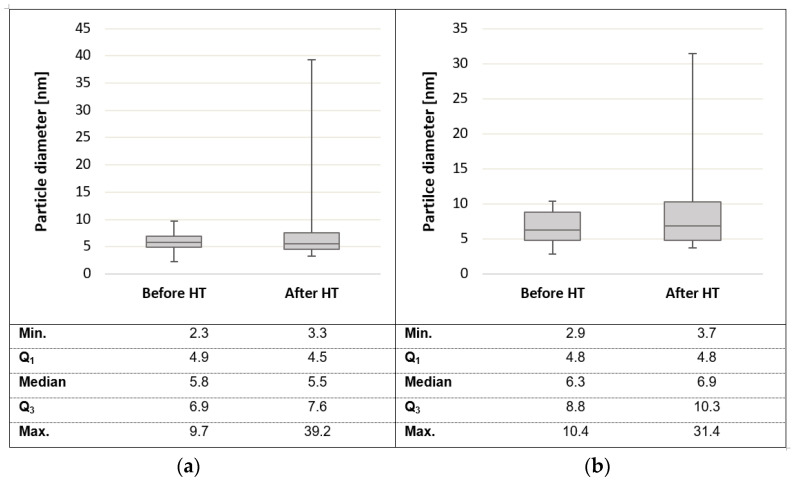
Box and whiskers diagram of the particle size distribution based on the particle diameters for (**a**) Pt and (**b**) Pt-Ru, before and after high-temperature operation (HT).

Transmission electron microscopy (TEM) images displayed in Figure 15 reveal that particle agglomeration/aggregation after HT application could have caused the decrease in the ECSA. The scale sizes of the different TEM images are not the same (scales are indicated in the lower left corners of each image), and hence the images are not indicative of the exact size difference; rather, they provide an indication of the agglomeration/aggregation evident after heat exposure. Figure 16 shows the particle size distribution before and after heat exposure (where ‘high-temperature exposure’ is referred to as HT in the figure).

Figure 15a,d (TEM images before HT) shows that there was almost no aggregation of particles, although major particle size differences were evident. The individual particles can clearly be seen (the darkened sections indicate overlapping particles). Most of the particles in (d) appear to be spherical, whereas the particles in image (a) show odd shapes. The porosity of these particles could be seen (light particles) but could not be quantified by TEM. The Pt and Pt-Ru nanoparticle aggregates are observed in (b), (c), (e), and (f). The extent of the particle agglomeration differs in the four images. The shape of the particles changed from small spherical to larger elongated particles. The most severe aggregation is seen in (b), where irregular particle lumps were formed. 

In Figure 16, a box and whiskers diagram of the particle size distribution (based on the particle diameter) for both Pt/C and Pt-Ru/C, before and after heat exposure, is given. A very similar particle size distribution is seen for both Pt and Pt-Ru, before heat treatment. The average particle sizes for Pt and Pt-Ru were ~5.8 nm and ~6.5 nm, respectively.

The Pt particles before HT application were in the range 2.3–9.7 nm, whereas the Pt-Ru particle sizes before HT experiments were in the range 2.9–10.4 nm. After HT experiments, the minimum particle size increased by 1 nm for Pt and by 0.8 nm for Pt-Ru. The values for Q_1_ (upper boundary for quartile one) and the median were very closely related before and after HT operation for both Pt and Pt-Ru. The Q_3_ values increased by 0.7 and 1.5 nm for Pt and Pt-Ru, respectively. There was a large difference in the maximum particle size (max.); the maximum particle size before HT operation changed from 9.7 and 10.4 nm to 39.2 and 31.4 nm after HT for Pt and Pt-Ru, respectively. This means that, for the Pt-Ru particles, almost a quarter of the heat-treated particles were larger than the maximum particle size before HT exposure.

Moreover, for Pt, the average particle size before HT operation was 5.7, and it changed to 7.35 nm after HT operation. Similar results were seen for Pt-Ru, where the average particle sizes changed from 6.53 nm to 8.91 nm. Hence, the aggregation of the nanoparticles was confirmed. It is known that the aggregation of particles has an impact on the surface area, as larger particles reduce the available surface, therefore resulting in lower ECSA values. This could be explained by the reduction in ECSA seen in Table 3. However, this was only considered to influence the ECSA at higher temperatures. CO poisoning is expected to be the greatest contributor to loss of ECSA at LT. It remains uncertain, however, as to at what temperature the particle agglomeration commences. Overall, it was concluded that analysis of the ECSA is more complex at HT than initially thought. No further attention was given to explaining the reduction in ECSA.

## 4. Considerations and Future Work

Due to the novelty of the work presented and some limitations identified within the study, some aspects should be addressed and considered in future work to clarify and support the data reported here. A summary of the aspects to consider is given below. 

A possible concern that could be raised is the high current values that were obtained in the CV scans, given that similar experiments normally yield currents in a range much lower than the reported data. However, this can be explained by the difference in in situ and ex situ application as mentioned before. Typical CV experiments are usually performed in an ex situ three-electrode setup. As reported by Garrick et al. [22], results for in situ and ex situ vary greatly. Garrick et al. [22] also reported high current CVs in a similar range as the data reported here. To verify and also to quantify the difference in in situ and ex situ CVs for the current experiments, the authors suggest for future work to, in parallel, repeat the experiments for an ex situ three-electrode RDE and an in situ MEA and to compare the results under the same conditions. Parameters that could possibly influence the results obtained for the in situ measurements should also be considered in the study. These include parameters such as the flow field design and setup, construction material and heat distribution of the flow fields, the nature of the catalyst (e.g., porous or nonporous, catalyst loading, particles (shape and size) or sheets, particle agglomeration), the contact resistance of the cell components (the assembly torque of the back plates), and the doping level and leaching of the acid.

In terms of Pt-Ru, it is possible for this alloy to undergo corrosion and dealloying (where Ru could dissolve into the membrane) at the given conditions. This phenomenon should be investigated in future work to verify if this is an issue and also to quantify the extent of the phenomenon and the effect it has on the measured values. 

Another limitation of the study that should be addressed in future work is the complex coupling chemistries that occur between CO and H_2_. It is recommended that the same COS tests be performed with CO/N_2_ as anode feed gas mixture to evaluate the CO tolerance in the absence of the coupling chemistries brought about by H_2_. These results could then be used to verify the results presented in this report. The authors also suggest that future research should include CV scans together with an analytical method, such as gas chromatography (GC) analysis, under the same operational conditions, to try to identify the products formed from the coupling chemistries between H_2_ and CO. This can be done with different concentrations of H_2_/CO. However, it should be taken into consideration that, if the operating temperature is increased, improved reaction will result, and this may cause immediate conversion from one chemical component to another. Therefore, GC analysis may not include all the reactions that are taking place on the Pt surface. 

## 5. Conclusions

This article describes the CO tolerance of Pt/C and Pt-Ru/C electrocatalysts in the temperature range 80–160 °C, evaluated using CV. Three types of CV peaks were considered to evaluate the ECSA: the COS-based ECSA, the H_upd_-based ECSA, and the H_upd_/COS-based ECSA. Both membranes were PBI-based with anode catalyst loading of 2 mg cm^−2^ Pt/C and Pt-Ru/C, respectively. The cathode electrocatalyst for both MEAs was Pt/C, and the active membrane area for the membranes was 25 cm^2^, with a thickness of approximately 58.90 μm (determined).

For the H_upd_-based ECSA evaluation, it was found that the ECSA is temperature-dependent and increases with an increase in temperature, for both Pt/C and Pt-Ru/C. The COS CV scans, performed with a 2% CO (balance H_2_) gas mixture, showed that both Pt and Pt-Ru were deactivated by CO at 80 °C; therefore, the ECSA was not evaluated at this temperature. Moreover, Pt was seen to be strongly affected by the coupling chemistries existing between CO and H_2_. These overlapping mechanisms were so significant that the ECSA could not be evaluated by the H_upd_/COS-based method. Since no CO peaks were seen (whereas other peaks were seen), COS could also not be used to analyze the ECSA. However, upon the addition of Ru to Pt, the ‘side’ reactions brought about by H_2_ and CO were suppressed, and H_upd_/CO peaks were observed for Pt-Ru. At temperatures ≥140 °C, no side reactions were seen. Only H_upd_/COS peaks were visible. This is what was expected for Pt, since CO should not adsorb onto the surface of Pt at temperatures >140 °C. Hence, the conclusion was drawn that Pt-Ru is ultimately more tolerant to the coupling chemistries existing between CO and H_2_. 

The ECSA results obtained from the H_upd_ peaks were compared with those obtained from the H_upd_/COS peaks. The H_upd_/COS-based values were lower. This is explained by the CO poisoning of the catalyst at LT. Since no peaks other than the H_upd_/COS peaks are visible for the COS scans performed on Pt-Ru at temperatures ≥140 °C, CO poisoning is not expected to influence the ECSA at temperatures >140 °C. TEM images revealed that particle agglomeration takes place after heat exposure. This is considered to be the cause of the lower ECSA values at HT. The TEM images were recorded after the CV experiments were carried out, and hence it is uncertain at what temperature the particle agglomeration commences. 

To conclude, it is well known that CO alters the performance of Pt-based electrocatalysts and that Ru is known to alleviate the poisoning effect that CO has on Pt at low temperatures. However, our studies now reveal that Ru serves to be valuable for CO mitigation, even at high temperatures (>100 °C). The utilization of Pt-Ru could be a possible solution for keeping active sites free for hydrogen pumping at temperatures >100 °C. However, this technique will require further research to validate its effect, to determine the optimal operating conditions, and to quantify the extent of the alleviation of CO poisoning. 

## Figures and Tables

**Table 1:** Pt/C Hupd-based ECSA estimation data at different temperatures, determined via the standard baseline method.

Temp. (°C)	Q_H_ (C)	A_EC_ (cm^2^)	ECSA (cm^2^ mg_Pt_^−1^)
80	7.39 ± 0.05	35,190 ± 240	704 ± 5
100	10.56 ± 0.05	50,286 ± 240	1006 ± 5
120	10.82 ± 0.05	51,524 ± 240	1030 ± 5
140	11.03 ± 0.05	52,524 ± 240	1050 ± 5
160	12.28 ± 0.05	58,476 ± 240	1170 ± 5

**Table 2:** Pt-Ru/C Hupd-based ECSA estimation data at different temperatures, determined via the standard baseline method.

Temp. (°C)	Q_H_ (C)	A_EC_ (cm^2^)	ECSA (cm^2^ mg_Pt_^−1^)
80	6.61 ± 0.05	31,462 ± 240	629 ± 5
100	9.96 ± 0.05	47,433 ± 240	949 ± 5
120	10.45 ± 0.05	49,762 ± 240	995 ± 5
140	11.00 ± 0.05	52,381 ± 240	1048 ± 5
160	12.11 ± 0.05	57,667 ± 240	1153 ± 5

**Table 3:** Pt-Ru/C (CO-H_upd_)-based ECSA estimation in terms of cm^2^ (mg catalyst)^−1^ (100–160 °C).

	100 °C	120 °C	140 °C	160 °C
Cycle 1	7 ± 0.5	166 ± 2	586 ± 5	624 ± 5
Cycle 2	161 ± 1	318 ± 3	632 ± 5	781 ± 5
Cycle 3	239 ± 2	654 ± 5	705 ± 5	874 ± 5

## Data Availability

Not applicable.
